# Identification of *Sarcocystis* and *Trichinella* Species in Muscles of Gray Wolf (*Canis lupus*) from Lithuania

**DOI:** 10.3390/vetsci11020085

**Published:** 2024-02-10

**Authors:** Evelina Juozaitytė-Ngugu, Evelina Maziliauskaitė, Muza Kirjušina, Petras Prakas, Rasa Vaitkevičiūtė, Jolanta Stankevičiūtė, Dalius Butkauskas

**Affiliations:** 1 Nature Research Centre, Akademijos 2, 08412 Vilnius, Lithuania; evelina.maziliauskaite@gamtc.lt (E.M.); prakaspetras@gmail.com (P.P.); dalius.butkauskas@gamtc.lt (D.B.); 2 Department of Ecology, Institute of Life Sciences and Technology, Daugavpils University, Parādes 1, 5401 Daugavpils, Latvia; muza.kirjusina@du.lv; 3 Agriculture Academy, Vytautas Magnus University, Studentų 11, Akademija, 53361 Kaunas, Lithuania; rasa.vaitkeviciute1@vdu.lt (R.V.); jolanta.stankeviciute1@vdu.lt (J.S.)

**Keywords:** *Sarcocystis*, *Trichinella*, gray wolf, molecular identification, host-specificity, phylogeny

## Abstract

**Simple Summary:**

The gray wolf (*Canis lupus*) is the largest representative of the family Canidae widespread in Eurasia and North America. *Sarcocystis* and *Trichinella* parasites were previously reported in the muscles of gray wolves. Apicomplexan *Sarcocystis* forms sarcocysts in the muscles of intermediate hosts and develops sporocysts in the intestines of definite hosts. Members of the genus *Trichinella* are cosmopolitan hazardous nematodes. The species composition of these parasites in gray wolves from Lithuania has not been studied so far. We examined muscle samples from 15 gray wolves, and species of parasites were confirmed using DNA analysis methods. Microscopically, *Trichinella* larvae were observed in 12 animals, and sarcocysts formed by *Sarcocystis* spp. were noticed in four. *Trichinella britovi* was also identified in the examined wolves. Current data show that zoonotic *T. britovi* is the dominant *Trichinella* species in gray wolves from nearby countries. In the case of *Sarcocystis*, two animals harbored *S. svanai*, and another two individuals were infected by *S. svanai* and *S. arctica*. Future studies are needed to assess the pathogenesis of the identified *Sarcocystis* spp.

**Abstract:**

Apicomplexan *Sarcocystis* and *Trichinella* nematodes are food-borne parasites whose life cycle is carried-out in various wildlife and domestic animals. The gray wolf (*Canis lupus*) is an apex predator acting as an ecosystem engineer. This study aimed to identify the species of *Sarcocystis* and *Trichinella* found in the muscles of gray wolves in Lithuania. During the 2017–2022 period, diaphragm, heart, and hind leg samples of 15 animals were examined. Microscopical analysis showed the presence of two types of *Sarcocystis* parasites in 26.7% of the analyzed muscle samples. Based on the sequencing of five loci, nuclear *18S* rDNA, *28S* rDNA, *ITS1*, mitochondrial *cox1*, and apicoplast *rpoB*, *S. arctica*, and *S. svanai* were identified. The current work presents the first report of *S. svanai* in gray wolf. Phylogenetically, *S. svanai* clustered together with *S. lutrae*, infecting various carnivorans, and *S. arctica* was most closely related to *S. felis* from domestic cats. *Trichinella* spp. were found in 12 gray wolves (80%). For the first time, *Trichinella* species were molecularly identified in gray wolves from Lithuania. *Trichinella britovi* was confirmed in all of the isolated *Trichinella* larvae using a multiplex PCR. Gray wolves in Lithuania may serve as a major source of zoonotic pathogens due to the presence of these parasites.

## 1. Introduction

Within the European Union (EU) (Large Carnivore Initiative for Europe 2022), the gray wolf (*Canis lupus*) population is estimated to be around 19,000 animals across the 27 EU Member States. In 2016, a population of 14,300 gray wolves was assessed [1]. Likewise, the gray wolf population grows in Lithuania. A 2021 report from the LIFE project shows that are about 504 wolves in the country. The gray wolf is a protected species in Europe according to the Council Directive 92/43/EEC on the Conservation of Natural Habitats and of Wild Fauna and Flora [1].

The gray wolf has the most extensive distributional range of any terrestrial mammal, encompassing North America, Europe, and Asia [2,3]. The animal can be found in many different places, such as deserts, grasslands, mountains, taiga, temperate forests, and arctic tundra [2,3,4]. This carnivore is the largest extant member of the family Canidae and is considered a habitat generalist, highly territorial, mobile, and has large individual territories [2,3,4,5]. *Canis lupus* is an apex predator species that indicates environmental health and plays a prominent role in any ecosystem they inhabit as ecosystem engineers [2,3,6]. The gray wolf primarily preys on red deer (*Cervus elaphus*), roe deer (*Capreolus capreolus*), elk (*Cervus canadensis*), American bison (*Bison bison*), wild boar (*Sus scrofa*), and other ungulates [2,3,4,7]. They also hunt small animals like beavers, rodents, and hares [2,8]. *Canis lupus* serves as a host for various parasites, including nematodes such as *Ancylostoma* spp. [9,10,11,12], *Capillaria*/*Eucoleus* spp. [9,11,12], *Trichinella* spp. [10,13], *Trichuris* spp. [9,11,12], *Toxocara* spp. [9,11,12], *Uncinaria* spp. [9,10,11,12], cestodes of *Echinococcus* spp. [7,10], *Taenia* spp. [9,10,11,12] and trematodes, as *Alaria alata* [9,10,11,12] and numerous unicellular organisms such as *Sarcocystis* spp. [14,15].

Zoonotic *Trichinella* spp. and *Sarcocystis* spp. can be found in the muscle tissue of gray wolves [10,13,14,15,16]. *Sarcocystis* (Apicomplexa: Sarcocystidae) and *Trichinella* (Nematoda: Trichinellidae) are worldwide-distributed parasites that infect mammals, birds, and reptiles [10,13,14,15,16,17].

Apicomplexan parasites of the genus *Sarcocystis* have an obligatory two-host life cycle based on a nutritional predator–prey relationship [15,16]. Asexual stages (merogony) develop only in the intermediate host (IH) (prey). During the stages of merogony and nuclear division, a motile merozoite forms [18]. Through the process of endodyogeny, banana-shaped zoites called bradyzoites are produced, which are located in the medullas of sarcocysts [19]. The IH acquires infection by ingesting food or water contaminated with excreted sporocysts. The sexual stages (gametogony) and sporulation of oocysts in the intestine evolve only in the definitive host (DH) (predator or scavenger) [18,19]. The DH becomes infected by consuming tissues harboring intracellular tissue cysts called sarcocysts [15,19]. *Sarcocystis* is a common genus of parasite in the Apicomplexa phylum, with over 200 known species [15]. Gray wolves usually act as DHs for numerous *Sarcocystis* spp. by producing sporocysts in their intestines [7,15,20]. However, this carnivore can also become an IH for *Sarcocystis* species. To date, only *S. arctica* has been described in the tongue muscles of the Alaskan wolf (*Canis lupus*) in 2016 [14]. Notably, it has been considered that *S. arctica* and *S. caninum*, described in the muscles of domestic dog (*Canis familiaris*), are the same species of *Sarcocystis*, and *S. caninum* is assumed to be a junior synonym of *S. arctica* [21,22].

Two *Sarcocystis* species, *S. arctica* and *S. lutrae*, have been identified in the muscles of Lithuanian carnivorans. Both of these species were detected in the hind leg muscles of the red fox (*Vulpes vulpes*) [23]. In addition, *S*. *lutrae* has been identified in the muscles of various mustelids, including the American mink (*Neovison vison*), the beech marten (*Martes foina*), the Eurasian badger (*Meles meles*), the Eurasian otter (*Lutra lutra*), and the European polecat (*Mustela putorius*) [24]. Until now, gray wolves have not been investigated as IHs for *Sarcocystis* parasites in Lithuania.

*Trichinella* nematodes have an exclusive life cycle, which contains two generations of parasites in the same host [25]. These parasites are released from larvae in the stomach after eating infected meat. The *Trichinella* larvae enter the intestinal lining, mature into adult stage, and then the adult males and females mate. Adult female worms release newborn larvae that can travel through the blood and lymphatic vessels in the body. Once the newborn larvae reach the striated muscle, they actively penetrate the muscle cells. The larvae mature inside infected host muscles (forming nurse cells) [26]. *Trichinella* parasites are circulated in two cycles maintained in nature, in domestic animals, for instance, in swine (*Sus scrofa domesticus*), horse (*Equus caballus*), and in sylvatic ones, for example, in the wild boar, the gray wolf, and the red fox [10,17,26,27,28]. In 2001, *Trichinella* species were confirmed in wolves using genetic methods in Estonia, Russia, and Spain [29,30,31]. Since then, these parasites have been extensively studied throughout Europe using multiplex PCRs [32]. In Lithuania, parasitological *Trichinella* spp. studies were conducted on wolves; however, species were not distinguished using molecular analysis methods [33].

The present study aimed to search for and identify *Sarcocystis* and *Trichinella* species in the muscles of gray wolves from Lithuania.

## 2. Materials and Methods

### 2.1. Sample Collection

**Figure 1:**
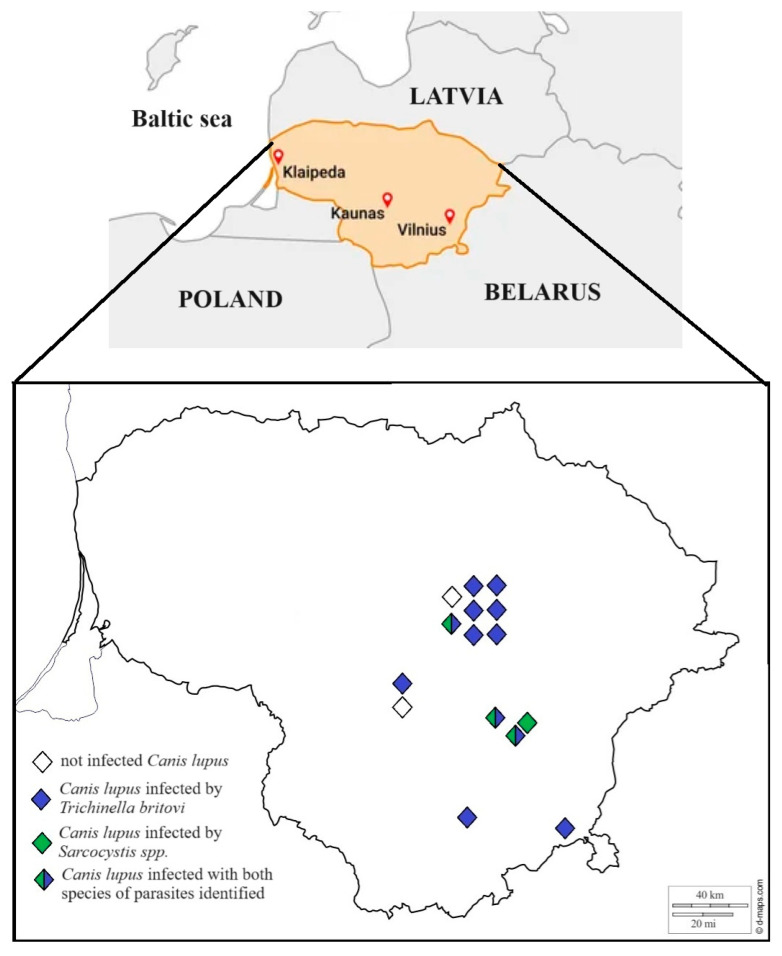
*Sarcocystis* spp. and *Trichinella* sp. in the gray wolf in Lithuania. The filled diamond shape represents positive individuals, and the empty diamond shape represents negative individuals.

Although gray wolves are protected throughout the EU by the Habitats Directive and the Bern Convention, limited hunting is permitted as long as it does not affect the conservation status of the population in Lithuania. The number of gray wolves hunted each season is set by the order of the Minister of Environment in Lithuania, and these mammals are hunted from October 15th to April 1st. In cooperation with local hunters, samples of muscle tissue (diaphragm, heart, and muscles of hind legs) were taken from 15 gray wolves and delivered to the Laboratory of Molecular Ecology, Nature Research Centre, Vilnius, Lithuania, for detailed morphological and molecular analysis of *Sarcocystis* spp. and *Trichinella* spp. No gray wolves were killed for the purpose of the present study. No permit was needed for the investigations in the current study, as stated by the requirements of the Minister of Environment in Lithuania. Samples were obtained between 2017 and 2022 from central and southern Lithuania (Figure 1). The muscle samples were stored frozen (at –20 °C) until further analysis.

### 2.2. Morphological Examination for the Presence of Sarcocystis spp. and Trichinella spp.

The presence of *Sarcocystis* spp. and the infection intensity of sarcocysts were evaluated in methylene blue-stained muscle samples. For this aim, 28 oat-sized pieces of muscle were cut off and stained with a water (1:500) and methylene blue solution. Later, muscle samples were lightened with 1.5% acetic acid solution, and pressed in a glass compressor consisting of 28 cross-sections. Subsequently, the morphological characterization of the sarcocysts and bradyzoites was conducted using freshly squashed muscle samples. Sarcocysts were removed with two preparation needles, measured using a computerized image analysis system, and put in a tube.

To detect *Trichinella*, each muscle sample was digested separately using a modified magnetic stirrer procedure, as described previously [34]. Notably, each organ (the diaphragm, heart, and muscles of the hind legs) was tested by artificial digestion separately. Then, 25% hydrochloric acid (16 ± 0.5 mL) was added to 1.5 L of tap water that was preheated to 46–48 °C in a 2 L glass beaker. In addition, 10 ± 0.2 g of pepsin was added to the acidic solution. In addition, 50 g of muscle tissue (the diaphragm, the heart, or the hind legs muscle) from one wolf was chopped up in a grinder. The digestive fluids were mixed for 30 min. This method is recognized by the European Food Safety Authority as the most effective method for detecting *Trichinella* spp. The infection intensity was estimated by counting *lpg* (the number of larvae per gram of sample). Microscopic examination was conducted as previously described by EURLP [35].

For the detection and characterization of sarcocysts, a Nikon ECLIPSE 8oi light microscope (Nikon Corp., Tokyo, Japan) was used, while morphological examination of *Trichinella* spp. was performed with the help of a Kern OZL-463 stereo microscope (Kern, Germany).

*Sarcocystis* spp. excised from fresh muscle samples of gray wolves and *Trichinella* spp. larvae collected from digested samples were preserved individually, in separate tubes containing 96% ethyl alcohol, and preserved at –20 °C for the molecular examination.

### 2.3. Molecular Analysis of Sarcocystis spp. and Trichinella spp.

DNA extraction of *Sarcocystis* sarcocysts was carried out with the GeneJET Genomic DNA Purification Kit (Thermo Fisher Scientific Baltics, Vilnius, Lithuania) in accordance with the manufacturer’s recommendations. For each individual infected with *Trichinella* nematodes, 10 larvae were analyzed by molecular tests. DNA extraction of *Trichinella* spp. was carried out according to the methodology of Pozio et al., 2003 [32]. Each *Trichinella* larvae was washed in PBS, placed with 5 μL of PBS, and added 2 μL Tris-HCl, pH 7.6. Then, the sample was heated at 90 °C for 10 min and cooled on ice for 10–15 min. Then, 9 μL of proteinase K solution was added (final concentration 100 μg/mL). The sample was incubated at 48 °C for 3 h and then the process of heating at 90 °C for 10 min repeated. In the end, samples of DNA were stored at –20 °C until use. The genomic DNA was extracted from single *Trichinella* larvae separately.

*Sarcocystis* species were characterized at five loci, *18S* ribosomal DNA (rDNA), *28S* rDNA, *ITS1* (internal transcribed spacer 1 region), *cox1* (mitochondrial gene encoding subunit 1 of cytochrome c oxidase), and *rpoB* (RNA polymerase B gene of the apicoplast genome). The nearly complete *18S* rDNA sequences, partial *28S* rDNA sequences, complete *ITS1* sequences, partial *cox1* sequences, and partial *rpoB* sequences were amplified using primers previously mentioned by Prakas et al., 2018 [36]. Each PCR mixture consisted of 25 μL containing 12.5 μL of Dream Taq PCR Master Mix (Thermo Fisher Scientific, Vilnius, Lithuania), 0.5 µM of both forward and reverse primers, 4-μL template DNA, and nuclease-free water. The PCR cycling conditions started with 5 min at 95 °C, followed by 40 cycles of 45 s at 94 °C, 60 s at 50–60 °C depending on the primer pair, and 80 s at 72 °C, and ended with 7 min at 72 °C. PCR products were evaluated using a 1% agarose gel, visualized via UV light after staining with 0.05 μg/mL ethidium bromide, and 5 µL of each PCR product was purified with alkaline phosphatase FastAP and exonuclease ExoI (Thermo Fisher Scientific Baltics, Vilnius, Lithuania) to remove unincorporated nucleotides and primers. Purified PCR samples were sequenced using a Big-Dye^®^Terminator v3.1 Cycle Sequencing Kit (Thermo Fisher Scientific, Vilnius, Lithuania) and a 3500 Genetic Analyzer (Applied Biosystems, Foster City, CA, USA) according to the manufacturer’s instructions. The identical forward and reverse primers used for the PCRs were used for both orientations of sequencing.

To calculate genetic similarity and choose *Sarcocystis* species for phylogenetic analysis, the DNA sequences from this study were compared with those of the Sarcocystidae family using Nucleotide BLAST [37]. For the phylogenetic study, sequences were aligned with the help of the MUSCLE algorithm implemented in MEGA7 [38]. The following software was used for the selection of nucleotide substitution models and the construction of phylogenetic trees based on the Maximum likelihood method. Taking into account the calculated lowest Bayesian Information Criterion values, T92+G+I was selected for *28S* rDNA and *rpob*, T92+G was chosen for *ITS1*, GTR+G+I was set for *cox1* and K2+G+I was selected for *18S* rDNA [39]. The bootstrap method with 1000 replications was used to test the robustness of the phylogeny.

*Trichinella* species were identified using the multiplex PCR technique as described previously [32,35]. The primer pairs used for species identification amplify the *ES5* (expansion segment 5) and *ITS1* (internal transcribed spacer 1) genetic regions (Supplementary File, Table S1) of the genus *Trichinella*, which encode ribosomal components [35]. PCR was performed following the conditions outlined in Supplementary File, Table S2. Electrophoresis was performed on a 2% agarose gel with ethidium bromide. Five µL of each obtained PCR sample and GeneRuler Low-Range DNA Ladder molecular mass marker (Thermo Fisher Scientific Baltics, Lithuania) were injected into the well. Electrophoresis was performed for 50 min using a 90 V electric current on a gel soaked in a 1× TAE buffer. After the procedure, the PCR products were visualized under UV light. The identification of *Trichinella* species was further confirmed by Sanger sequencing. For this purpose, amplified species-specific PCR products, generated using DNA of larvae isolated from each infected animal (*n* = 12), were excised from agarose gel using GeneJET Gel Extraction Kit (Thermo Fisher Scientific Baltics, Vilnius, Lithuania) and subjected to sequencing. Purified PCR products were sequenced bidirectionally as described above. The obtained sequences were compared with those of *Trichinella* spp. using Nucleotide BLAST [37].

### 2.4. Data Analysis

The prevalence of *Trichinella* spp. and mean *lpg* were calculated for examined muscle tissues in gray wolves individually. Bootstrap two-sample *t*-tests [39] based on 2000 replications were used to compare mean *lpg* values established in diaphragm and limb muscles. *p* < 0.05 was considered statistically significant. Statistical tests were carried out using the Quantitative Parasitology 3.0 software [40].

## 3. Results

### 3.1. Prevalence and Morphology of Sarcocysts of Sarcocystis spp.

Based on the methylene blue-staining, sarcocysts of *Sarcocystis* spp. were detected in 26.7% (4/15) of the gray wolf (Table 1). One animal (isolate ClLt10) had 58 and 45 sarcocysts in one gram of diaphragm and limb muscles, respectively. Other infected gray wolves harbored sarcocysts only in diaphragms (isolates ClLt3; ClLt8; and ClLt14). The average parasite load was 16.8 ± 27.6 sarcocysts/g of diaphragm (Table 1). Parasites were not noticed in the heart muscles.

**Figure 2:**
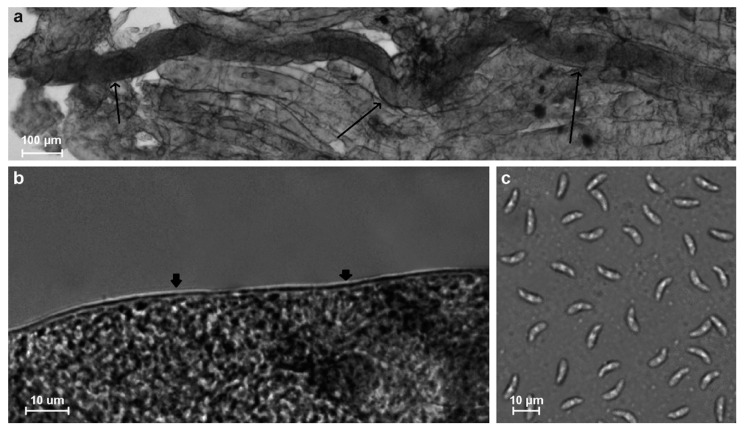
Morphology of *Sarcocystis svanai* from muscle tissue of a gray wolf. Light micrographs. Fresh preparations (**a**–**c**). A portion of the ribbon-shaped sarcocyst (shown by arrows) (**a**), thin cyst wall (arrows) (**b**), lancet-shaped bradyzoites (**c**).

In fresh samples, sarcocysts were detected in four animals. The sarcocysts found in two samples (isolates ClLt3 and ClLt8) were microscopic, ribbon-shaped, 950–1806 × 33–74 μm in size, with a thin (0.5–1.0 μm), apparently smooth cyst wall (Figure 2a,b). Bradyzoites were banana-shaped, 5.7–9.4 × 1.2–2.7 μm in size (Figure 2c). The DNA sequence analysis showed that these sarcocysts belong to *S. svanai* (Table 1).

**Figure 3:**
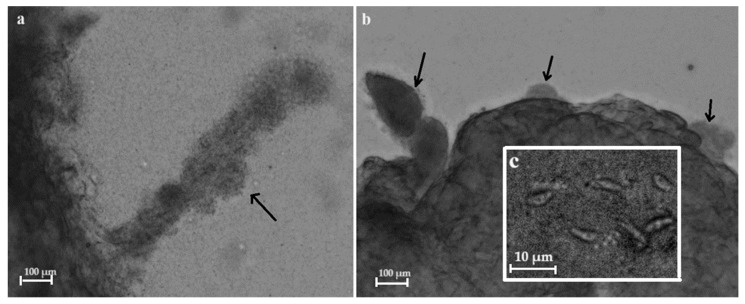
Sarcocyst remnants from muscle tissue of the gray wolf (shown by arrows). Light micrographs (**a**–**c**). The elongated shape of sarcocyst-like bradyzoite nodules from limb muscle (**a**), the nodules of bradyzoites from the diaphragm (**b**), magnified image of lancet-shaped bradyzoites visible in the nodules of bradyzoites (**c**).

In the other two samples (ClLt10 and ClLt14), sarcocysts with smooth cyst walls were found, along the remnants of sarcocysts. In particular, the cyst wall had disappeared, leaving only the cyst-shaped bradyzoite nodules that were visible in the sarcocyst remnants (Figure 3). A whole cut piece of muscle was used for the DNA extraction of *Sarcocystis* sp. Further molecular investigations revealed that these remnants of sarcocysts belong to *S. arctica* (Table 1).

### 3.2. Genetic Characterisation and Phylogeny of S. arctica and S. svanai

The PCRs and sequencing were successful for all six isolates in the five genetic loci examined, except for two *S. svanai* isolates in *ITS1*. From the current study, the generated 1781 bp *18S* rDNA, 1500 bp *28S* rDNA and 958 bp *ITS1* sequences of *S*. *svanai*, 1753 bp *18S* rDNA, 1461 bp *28S* rDNA and 697 bp *ITS1* sequences of *S*. *arctica,* 1053 bp *cox1*, and 762 bp *rpob* sequences of *S*. *svanai* and *S. arctica* are available in NCBI GenBank under the accession numbers OR921254–OR921265, OR935783–OR935786, and OR939976–OR939987. The obtained sequences of *S*. *arctica* were 100% identical in all five loci examined, whereas *S*. *svanai* differed by one single nucleotide polymorphism (SNP) in *ITS1*. The alignment of our sequences displayed indels (insertions/deletion) within *18S* rDNA, *28S* rDNA, and *ITS1*, while *rpob* and *cox1* sequences differed only by substitutions. A particularly large variation in length was observed when comparing the *ITS1* sequences of two *Sarcocystis* species identified. These two *Sarcocystis* species showed very high similarity within *18S* rDNA and *cox1* (99.4–99.5%), a slightly lower similarity within *28S* rDNA and *rpob* (97.9–98.5%), and even differences of 19.0% within *ITS1* (Table 2). Comparing intraspecific and interspecific differences estimated, it has been noted that *S*. *arctica* and *S*. *svanai* cannot be identified by the *cox1* fragment examined, whereas *ITS1*, *rpob,* and *28S* rDNA are most suitable for the discrimination of these species.

In this work, obtained *18S* rDNA sequences of *S*. *arctica* were 100% identical to *S*. *arctica* (KF601301, KY947306-7, KX022100-3, KX156838, MF596217-37, and MZ329343) and *S*. *caninum* (MH469238), 99.4% similar to *S*. *fulicae* (MG273671), *S*. *halieti* (JQ733511, MF946587, MH130211, MZ329386, MZ329390), *S*. *lari* (MF946588), *S*. *turdusi* (JF975681), and *S*. *wobeseri* (EU502869), using birds as their IHs and DHs [41,42]. Based on *28S* rDNA, *S*. *arctica* from the Lithuanian gray wolf were 100% identical to *S*. *caninum* (MH469239), demonstrated 99.9–100% similarity to *S*. *arctica* (KF601312, KY609323, KY947308-9, KX022104-7, MF596240-60), 99.4–99.5% similarity to *S*. *felis* (OR436907–OR436910) from the domestic cat, and 98.5% similarity to *S*. *lari* (JQ733509, MF946611). The *cox1* sequences of *S*. *arctica* shared 99.8–100% similarity compared to other isolates of this species (KF601318-21, KY609324, KY947304-5, KX022112-5, KX156839, MF596286-306, MZ332967); displayed 99.7–99.8% similarity to *Sarcocystis* sp. clone 1 (MW962266-9) from the black bear (*Ursus americanus*); 99.7% similarity to *S*. *caninum* (MH469240); 99.3–99.4% similarity to *S*. *lutrae* (KF601326, KM657808, MF596284-5, MG273661-70, MG372106-7, MT036250, MT036254, ON805825) circulating between predatory mammals of the families Mustelidae, Canidae and Procyonidae as IHs and birds as DHs [41,42]; and 97.3–99.4% similarity to multi-host adapted *S*. *canis* (KX721495-7) [43,44]. The *rpob* sequences of *S*. *arctica* from Lithuanian gray wolf showed 99.8–100% similarity to *S*. *arctica* (MF596311-21), 99.8% similarity to *S*. *caninum* (MH469242), 98.4% similarity to several *Sarcocystis* spp. (MF596307, MH138322, MH138325-6, LR884241), circulating between birds in their life cycle. Based on *ITS1*, the present study’s generated sequences of *S*. *arctica* were 100% identical to *S*. *arctica* (KF601306, KF601308, KY947310-1, KX022108-11, KX156837, MF596262-82, MZ333536, OK481372-6), had a 99.5–100% similarity to *S*. *caninum* (JX993923, MH469241), and an 88.0–96.3% similarity to *S*. *felis* (AY190081-2, MN508375-9, OQ676522).

The *18S* rDNA sequences of *S*. *svanai* from gray wolves in Lithuania disclosed 99.9–100% similarity to *S*. *svanai* (KM362428, KY292483-7), followed by up to 99.8% similarity to *Sarcocystis* spp., which use bird–bird hosts in their life cycle. Based on *28S* rDNA, *S*. *svanai* showed the greatest 98.3–98.5% similarity to *S*. *arctica* (KF601312, KY609323, KY947308-9, KX022104-7, MF596240-60), 98.4% similarity to *S*. *caninum* (MH469239), and 98.1–98.3% similarity to *S*. *lutrae* (KM657771-2, MF596238-9, MG272276-85, MG372104-5, MT036249, ON796572). At *cox1*, *S*. *svanai* was indistinguishable (100% identical) from *S*. *lutrae* (KF601326, KM657808, MF596284-5, MG273661-70, MG372106-7, MT036250, MT036254, ON805825) and *S*. *lari* (MF596283-4), and also showed high 99.8% similarity to some *Sarcocystis* spp. That employ birds as their hosts (MF946583, MH138308-9, MH138312, MH138314, MZ332968-9). Based on *rpob*, *S*. *svanai* from Lithuanian gray wolf were 100% identical to *S*. *svanai* (KC191640), displayed 98.8% similarity to *S*. *lutrae* (MF596309-10) and 98.4% similarity to *S*. *campestris* (GQ851963) from Richardson’s ground squirrel (*Spermophilus richardsonii*). The *ITS1* sequences of *S*. *svanai* demonstrated 75.0% similarity to *Sarcocystis* sp. CRC-836 (HQ184185) from the sperm whale (*Physeter macrocephalus*), 72.8–74.9% similarity to *S*. *lutrae* (KM657773-805, MF596261, MG272296-305, MG372108-9, OK481377, ON806939) 74.1% similarity to *S*. *kalvikus* (GU200661) from the wolverine (*Gulo gulo*), and 73.9–74.1% similarity to *Sarcocystis* sp. (MH918015, MW264422) from the subantarctic fur seal (*Arctocephalus tropicalis*).

**Figure 4:**
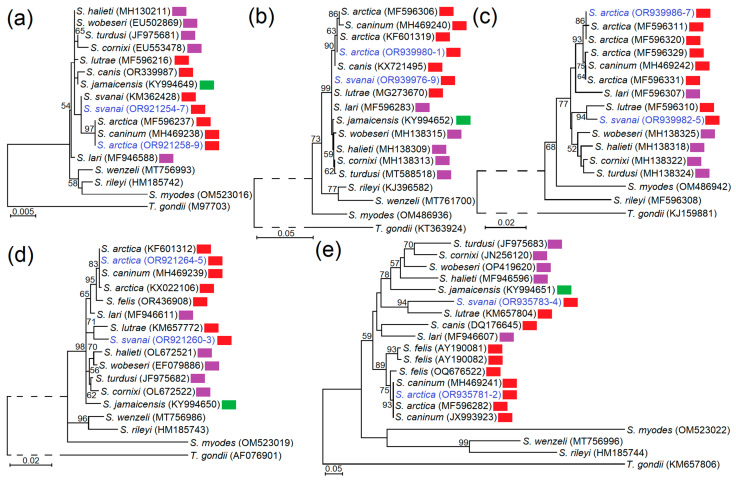
The phylogenetic placement of *S. arctica* and *S. svanai* isolated from gray wolves in Lithuania on the basis of *18S* rDNA (**a**), *cox1* (**b**), *rpob* (**c**), *28S* rDNA (**d**), and *ITS1* (**e**). Phylogenetic trees were constructed using Maximum Likelihood method, scaled according to branch length and rooted on *Toxoplasma gondii*. The dashed line points out that its length does not correspond to the evolutionary distance. Figures next to branches display bootstrap support values. Sequences obtained in the present work are highlighted in indigo. Red rectangles show *Sarcocystis* species using members of order Carnivora as their intermediate hosts. *Sarcocystis* species employing birds in their life cycle are indicated with purple rectangles and *Sarcocystis* species cycling between rodents and birds are indicated with green rectangles.

Two *Sarcocystis* species were identified in Lithuanian gray wolves clustered in phylogenetic trees together with other isolates of the same species (Figure 4). Our phylogenetic examination confirmed that *S*. *arctica* cannot be genetically differentiated from *S*. *caninum* in the five genetic loci studied. The phylogenetic analysis showed that *S*. *arctica* and *S*. *svanai* were placed together with *Sarcocystis* species using mammals of the order Carnivora as their IH (*S*. *canis*, *S*. *caninum*, *S*. *felis* and *S*. *lutrae*) and to species employing birds as their IHs and DHs (such as *S*. *cornixi*, *S*. *halieti*, *S*. *lari*, *S*. *turdusi*, *S*. *wobeseri*). Based on *rpob*, the *28S* rDNA, *ITS1*, *S*. *svanai* was a sister species to *S*. *lutrae*. In the phylogenetic trees, generated using *28S* rDNA and *ITS1* sequences, *S*. *arctica* was most closely related to *S*. *felis*.

### 3.3. Microscopical and Molecular Examination of Trichinella spp.

**Figure 5:**
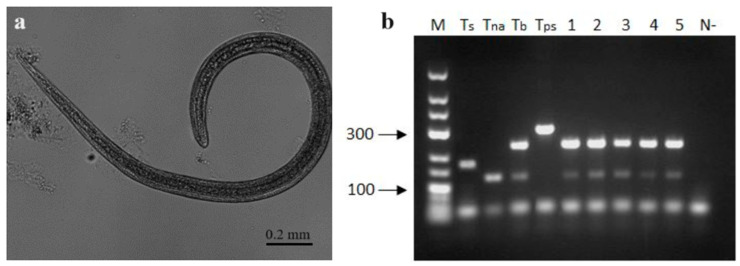
Morphological and molecular examination of *Trichinella* spp. in gray wolf. (**a**) *Trichinella* spp. larvae found by the method of artificial digestion in the diaphragm. (**b**) Agarose gel electrophoresis (2%) of multiplex PCR of *Trichinella britovi* in 5 larvae. M—“GeneRuler Low Range DNA Ladder” molecular weight marker, sizes are in base pairs, positive controls of: Ts—*T. spiralis*, Tna—*T. nativa*, Tb—*T. britovi*, Tps—*T. pseudospiralis*, 1–5—individual larvae from one sample, and N—Negative control.

Out of the 15 tested gray wolves, *Trichinella* spp. larvae (Figure 5a) were detected in 12 animals (80.0%). Two wolves (No. 1 and No. 2) were excluded from the statistical analysis of *lpg*, since it was not possible to check the diaphragms of these wolves and the intensity of *Trichinella* spp. infection in the hind legs of these animals was relatively high (Table 1). The intensity of *Trichinella* infection varied between 0.2 and 17 *lpg* in the diaphragm and 0.7 and 21 *lpg* in the muscles of the hind legs. The mean larval burden was not significantly different between two muscle samples ($\bar{x}$ = 5.79 ± 5.6 in the diaphragm and $\bar{x}$ = 5.63 ± 7.3 in the limb, *p* = 0.9790). No *Trichinella* parasites were detected in the heart muscle of gray wolves in present study.

*Trichinella* spp. larvae from 220 isolates were successfully identified at the species level by multiplex PCR. All analyzed muscle samples contained *Trichinella britovi* (Figure 5b), and no instances of species co-infection were observed. The PCR results were then confirmed with sequencing data. Twelve 253 bp *ITS1* sequences obtained from larvae isolated from all 12 infected gray wolves were 100% identical and were submitted to GenBank under accession number PP153335. These sequences from the Lithuanian gray wolf were 100% identical to sequences of some isolates of *T. britovi* (OK483203, OK483205-7, OK483214-5, KU374878-9, KU374883-4), 98.1–99.6% similarity to sequences of other isolates of *T. britovi* (OK483202, OK483204, OK483208-13, OK483216, KU374867, KU374875, KU374877, KU374881, KU374885), 97.3–98.4% similarity to those of *Trichinella murrelli*, 96.8% similarity to those of *Trichinella nativa* (KP307962-6), and 95.7–96.4% similarity to those of *Trichinella* sp. T6 (KP307967-71).

## 4. Discussion

### 4.1. Pathogenic Impact of Parasites Identified in Gray Wolf

The gray wolf is a “keystone species” that plays a vital role in maintaining the health, structure, and balance of ecosystems [2,3,4,5,8]. Gray wolves may spread more than 10 viral, bacterial, and mycotic diseases and more than 70 species of helminths and protists [9,10,11,12]. In the present study, sarcocysts of *S. svanai* were identified for the first time in gray wolves. Furthermore, *Trichinella britovi* and *Sarcocystis arctica* were for the first time confirmed in gray wolves in Lithuania.

Some *Sarcocystis* spp. may be pathogenic for IH [15]. To date, at least three pathogenic *Sarcocystis* species have been reported in canids, *S. caninum*/*S. arctica*, *Sarcocystis canis*-like, and *S. neurona* [15,23,43,45,46,47,48]. Sarcocysts morphologically similar to *S. caninum*/*S. arctica* have been reported in the muscles of two dogs from the USA [48] and in a dog from Canada [47] which suffered from severe myositis. In addition, more severe symptoms such as ataxia, stiff gait or inability to walk, generalized pain, anorexia, diarrhea, fever, and panting were retrieved in four dogs from the USA caused by *S. caninum*/*S. arctica* [49]. Later, of the eight reported cases of muscular sarcocystosis in dogs, five were related to clinical signs [15]. A fatal *S. caninum*/*S. arctica* and *S. svanai* coinfection revealed severe monophasic multifocal myodegeneration with severe pyogranulomatous inflammation in a dog reported from Finland [50]. Infections with highly pathogenic and multi-host-adapted *S. canis*-like and *S. neurona* have also been reported in dogs [47,51]. Thus, comprehensive investigations into the pathogenesis of *S. arctica* and *S. svanai* in canids are needed.

*Trichinella* nematodes cause a serious, and sometimes fatal, human disease called trichinellosis, which is a food-borne zoonotic disease with worldwide distribution [25,26,27,28]. Frequently, humans become infected with these parasites by eating raw or undercooked meat from infected animal products. In general, domestic swine and related products continue to be the most significant source of human *Trichinella* infection [26]. However, cases of trichinosis in humans have been recorded when the main source was game meat, such as wild boar [52], brown bear (*Ursus arctos*) [53,54], badger [54], walrus (*Odobenus rosmarus*) [55], and cougar (*Puma concolor*) [56]. Human symptoms of parasitic infections vary depending on the type of parasite, the level of infection, and the host’s immune response [57]. The life cycle of *Trichinella* in humans or animals follows three stages: the enteral phase (intestinal disorders), the parenteral phase (allergic reactions, myalgia, and fever), and the encysting phase (recovery) [58]. *Trichinella* larvae can survive in the muscles of their hosts for years, depending on the adaptations of the species. Even though parasites of this genus cause various symptoms in humans, *Trichinella* larvae do not appear to be pathogenic to other hosts (wild, domestic, or synanthropic animals) unless in large numbers in muscle [59]. While *T. spiralis* is known to be the most pathogenic species of *Trichinella* in humans, *T. britovi* is the second species of greatest concern. About 80% of people infected with *T. britovi* had myalgia, weakness, and arthralgia, about 70% experienced headaches, fever, and edema, and 20% had gastrointestinal disorders [60]. Also, *T. britovi* is one of the greatest concerns because it has some resistance to low temperatures and can survive in the host muscle for up to 6 months at a temperature of −20 °C [61].

### 4.2. Host Specificity of Sarcocystis Species from Canids

In this study, we identified *S. svanai* in gray wolves for the first time. Previously, this *Sarcocystis* species was detected in the muscles of two domestic dogs from the USA [21] and in the muscles of 19 Pampas foxes (*Lycalopex gymnocercus*) from Argentina [62]. Based on histopathological analysis, *S. svanai* was potentially also identified in one dog from Finland [50], whereas *S. arctica* was described in the muscles of two Arctic foxes (*Vulpes lagopus*) from Norway in 2014 [63]. Subsequently, this species was recorded in the muscles of one Alaskan wolf (*Canis lupus*) in 2016 [14], nine Arctic foxes from Alaska in 2017 [64], three red foxes from the Czech Republic in 2017 [65], and ten, two, and three red foxes from Latvia, Lithuania, and Spain in 2018 [23]. To date, at least four *Sarcocystis* spp., *S. arctica*/*caninum*, *S. canis*-like, *S*. *lutrae*, and *S. svanai* have been described in predatory mammals of the family Canidae [15,21,23,62,63,64,65]. Furthermore, dogs may serve as an aberrant dead-end host for highly pathogenic *S. neurona* [49]. *Sarcocystis vulpis*, found in the muscles of the red fox, is considered to be a species of inquirendae [15]. Some authors do not list *S*. *corsaci*, found in the corsac fox (*Alopex corsac*), as a valid species due to a lack of molecular data on this parasite [63]. Most of the *Sarcocystis* species are generally host-specific for their IHs [15]. The host specificity of *S. arctica* and *S. svanai* found in this study is restricted to the family Canidae. Meanwhile, *S*. *lutrae* has been identified in the muscles of three Carnivora families, Canidae, Mustelidae, and Procyonidae [23,24,66,67,68,69]. Asexual stages of *S. canis* have been identified in seven different mammalian families (Canidae, Chinchillidae, Delphinidae, Equidae, Otariidae, Phocidae, and Ursidae) [43,45,49]. In summary, further comprehensive investigations of the *Sarcocystis* spp. specificity for their IHs are required.

### 4.3. Morphological and Molecular Characteristics of Identified Sarcocystis Species

In the present study, two *Sarcocystis* species, *S. arctica* and *S*. *svanai* may be identified by clearly different sarcocyst wall appearances. It has been shown that the sarcocyst wall of *S. arctica* has short knob-like or dome-shaped protrusions, approximately 1–1.5 μm wide and 0.5–1 μm long [23,63,64,65], while the cyst of *S*. *svanai* is thin-walled (Figure 2b) [21]. However, in the present study, *S*. *arctica* sarcocysts were not detected; only cyst remnants and bradyzoites were visible (Figure 3). Freezing of gray wolf muscles may have adversely affected the sarcocyst structure of *S*. *arctica*. Similar observation issues have also been noticed in other studies on *Sarcocystis* spp. [15,62]. The freshness of muscle samples is therefore very important for the morphological analysis of sarcocysts and identification of *Sarcocystis* species.

In this study, we have, for the first time, genetically characterized *S*. *svanai* in *28S* rDNA, *ITS1* and *cox1*, as previously only *18S* rDNA and *rpoB* sequences of this species were available [21,62]. *18S* rDNA and *rpoB* sequences of *S*. *svanai* from gray wolf were 100% identical to *S*. *svanai* from domestic dog [21] and based on *18S* rDNA our sequences differed by one SNP (A/C) compared to *S*. *svanai* from Pampas fox (*Lycalopex gymnocercus*) [62]. Phylogenetic results showed that *S*. *svanai* clustered together with *S*. *lutrae* (Figure 4), while *S*. *arctica* was most closely related to *S*. *felis*. The noticed phylogenetic grouping is in agreement with morphological similarities of sarcocysts of species analyzed. Sarcocysts of *S*. *svanai* and *S*. *lutrae* are characterized by transmission electron microscopy as having 1a sarcocyst wall type [21,23,62], while sarcocysts of *S*. *arctica*/*S*. *caninum* and *S*. *felis* had similar 9c and 9a cyst wall types, respectively [15,63].

Based on the compiled data, *S*. *arctica* showed intraspecific variation within four genetic loci, *28S* rRNA, *ITS1*, *cox1* and *rpob* (Table 2). Previously, it was suggested that two genetic lineages of *S*. *arctica* distinguished by *cox1* are diverging along the latitudinal cline [23]. Such an assumption was proposed, since only *cox1* haplotype A was identified in gray wolf and arctic fox in Alaska, whereas the haplotype B was found in domestic dog in China and in red fox in Spain, and finally both haplotypes were present in red fox from the Czech Republic and from the Baltic States [23,65]. Here we also determined haplotype A for *S*. *arctica* isolated from the gray wolf in Lithuania.

Among the five examined loci, the identified *Sarcocystis* species had the highest variation within *ITS1*, followed by *28S* rDNA and *rpob* (Table 2). However, at *28S* rDNA and *rpob* interspecific similarity compared to most related species were still high, exceeding 98%. The genetic variability was very low in *18S* rDNA and *cox1*. These genes were not suitable for accurately differentiating the studied *Sarcocystis* species (Table 2, Figure 4). The results of our study complement previous investigations showing little value of *18S* rDNA and *cox1* in discrimination of *Sarcocystis* spp. employing Carnivora as their IH [23,63,64,65]. Notably, these two genes are mostly used for the identification of numerous *Sarcocystis* species with ungulates as their IHs [70]. In summary, due to the small genetic variability of *Sarcocystis* species parasitizing carnivorous mammals in *18S* rDNA, *28S* rDNA, *cox1* and *rpoB*, other genes need to be found for further detailed genetic characterization of these *Sarcocystis* species.

### 4.4. Prevalence and Species Composition of Trichinella in Gray Wolf

The identified high percentage of positivity of *Trichinella* spp. infection in gray wolf from Lithuania (80.0%) corresponds to high infection rates established in this host in Latvia (69.7–100%), Estonia (50–79.4%), and Russia (97.3%) [10,29,71,72,73]. Lower values of prevalence of *Trichinella* parasites in gray wolf had previously been documented in the Central Balkans (46.5%) [74], Finland (33.33–39.2%) [75], Croatia (31%) [76], Poland (54.5%) [77], Romania (31–40%) [78], Alaska (28–36%) [79], Italy (27.1%) [80], and the Western part of the Italian Alps (11.53%) [81]. In the current study, no significant differences in lpg were observed in the diaphragm (0.2–17; $\bar{x}$ = 5.79 ± 5.6) and limb (0.7–21; $\bar{x}$ = 5.63 ± 7.3) muscles. Similarly, relatively high lpg values varying between 0.009 and 27 lpg, 0.1 and 41.8 lpg, and 0.01 and 44.9 lpg were estimated in infected gray wolves from Poland [77], Latvia [73], and Estonia [29], respectively. The high infection prevalence and intensity of *Trichinella* spp. have been attributed to inappropriate hunting practices, such as not burying the carcasses of hunted animals or the use of meat for animals baiting [30]. In accordance with the considerations set out above and as established by EU legislation, it is also important that systematic testing and monitoring for *Trichinella* be carried out in all slaughtered pigs, wild boar and horses. Appropriate rodent control campaigns are also necessary. Using control procedures and protocols is important for ensuring the safety of food for consumers and monitoring the health of wild animals. The safety of meat should always be a top priority, regardless of its intended use. Additionally, improving hunter training (good slaughtering practices and proper hunter handling) is essential.

Currently, 10 *Trichinella* species (*T. spiralis*, *T. nativa*, *T. britovi*, *T. murrelli*, *T. nelsoni*, *T. patagoniensis*, *T. chanchalensis*, *T. pseudospiralis*, *T. papuae*, and *T. zimbabwensis*) and three genotypes (*Trichinella* T6, *Trichinella* T8, and *Trichinella* T9) are known worldwide [82]. Four species of the genus *Trichinella*, *T. britovi*, *T. nativa*, *T. pseudospiralis*, and *T. spiralis*, are found in Europe [83]. In the current study, *T. britovi* was identified in all 220 isolated larvae. Among other *Trichinella* species, *T. britovi* has the widest geographical distribution. High infection rates of *T. britovi* were reported in different carnivore families, Canidae, Felidae, and Ursidae, in various European countries [84]. Notably, *Trichinella* species in Lithuanian gray wolves have not been previously investigated by molecular methods. However, based on the examination of other wild canids sampled in 2000–2002 in the Baltic States, a high prevalence of *T. britovi* was recorded in red foxes (89.8%) and raccoon dogs (91.3%) from Lithuania and in raccoon dogs (91.3%) from Latvia [33]. In 2016, *T. britovi* was the most common species among studied wild predators in Latvia, accounting 94% [73]. A single *T. nativa* (1.1% infection) and two different mixed infections of *T. nativa*/*T. britovi* (4.4%) and *T. spiralis*/*T. britovi* (0.5%) were also detected in this study. Based on this study, *T. britovi* was found to be 100% common among gray wolves [73]. Previous similar surveys in Poland also showed that *T. britovi* was the main species in wolves [77]. *Trichinella britovi* is likely the most common species among gray wolves and other wild predators in neighboring countries [29,30,33,73,77]. The high prevalence of *T. britovi* infection may indicate that gray wolf may be an important contributor to the sylvatic cycle maintenance of this hazardous nematode. One hypothesis is that wild boar may serve as a natural reservoir of *Trichinella* infection for carnivorous [52]. For comparison purposes, in 2001 the distribution of *Trichinella* spp. among wild boars in Lithuania was 1.3% [85]. In 2019, the National Food and Veterinary Risk Assessment Institute of Lithuania found that 0.5% (43 out of 9200) of wild boars were infected with *Trichinella* larvae [86]. There is a lack of data on *Trichinella* infection rates in wild carnivores and omnivores in Lithuania to document the impact of wild boar on the spread of this disease. The reality is that the infection of wild boar with *Trichinella* spp. may be higher than the calculated rates. Another reason for the high percentage of *Trichinella* infection in predators studied could be the fact that the gray wolf population, which is rapidly growing as mentioned in the introduction, scavenges and cannibalizes more often. Also, our study raises the idea that humans influence the high percentage of *Trichinella* infection due to improper hunting practices as it has been shown in Russia [30].

## 5. Conclusions

In the present study, a new host record, i.e., gray wolf was provided for *S*. *svanai*. Furthermore, it was demonstrated that gray wolves from Lithuania are also infected with *S*. *arctica*. It was revealed that the muscles of a single gray wolf could be infected with two *Sarcocystis* species. Among five loci studied, *ITS1*, *28S* rDNA and *rpob* were most valuable for the genetic identification, and phylogeny of *Sarcocystis* species detected. Moreover, *T. britovi* was genetically confirmed in all isolated *Trichinella* larvae in the muscles of gray wolf for the first time in Lithuania. In wildlife, carnivore species such as the gray wolf may be an important reservoir of *Sarcocystis* spp. and zoonotic *Trichinella* spp. in Lithuania.

## Figures and Tables

**Table 1:** Presence, intensity, and molecular species identification of studied parasites in muscle samples of gray wolves from Lithuania.

No.	*lpg*		*Trichinella* Genotype	*Sarcocystis* spp.		*Sarcocystis* Species
	Diaphragm	Limb		Diaphragm	Limb	
1	No data	46	T3	-	-	
2	No data	16	T3	-	-	
3	12.04	2.26	T3	7	-	*S*. *svanai*
4	-	-	-	-	-	
5	2.24	0.96	T3	-	-	
6	-	-	-	-	-	
7	0.42	17.58	T3	-	-	
8	0.2	3.58	T3	1	-	*S*. *svanai*
9	6.7	1.96	T3	-	-	
10	0.64	0.7	T3	58	45	*S*. *svanai* and *S*. *arctica*
11	3.32	4	T3	-	-	
12	6.24	2.5	T3	-	-	
13	9.06	1.74	T3	-	-	
14	-	-	-	1	-	*S*. *svanai* and *S*. *arctica*
15	17	21	T3	-	-	

**Table 2:** The genetic comparison of *Sarcocystis* species identified in gray wolf from Lithuania.

Genetic Loci	*S. arctica*/*S. caninum* ^a^		*S*. *svanai*		Differences between *S. arctica* and *S. svanai*
	Intraspecific Differences ^b^	Interspecific Differences	Intraspecific Differences ^b^	Interspecific Differences	
*18S* rRNA	0	≥0.5	0.1	≥0.2	0.5
*28S* rRNA	0–0.1	≥0.5	0 ^c^	≥1.5	1.5–1.7
*ITS1*	0–0.5	≥3.7	0 ^c^	≥25.0	19.0 ^d^
*cox1*	0–0.3	≥0.2	0 ^c^	≥0	0.6
*rpob*	0–0.2	≥1.6	0	≥1.2	2.0–2.1

^a^ In the comparison *S*. *arctica*/*S*. *caninum* were considered as the same genetic species; ^b^ comparing in the present study obtained sequences with those of the same species available in GenBank; ^c^ prior to this investigation no *28S* rRNA, *ITS1*, and *cox1* sequences of *S. caninum* were available; ^d^ Query coverage was 82%.

## Data Availability

The sequences of *S*. *arctica*, *S*. *svanai*, and *Trichinella britovi* generated in the current research were submitted to the NCBI GenBank database. The *18S* rDNA, *28S* rDNA, *ITS1*, *cox1* and *rpoB* sequences of two *Sarcocystis* species are available under accession numbers OR921254–OR921259, OR921260–OR921265, OR935783–OR935786 and OR939976–OR939981, OR939982–OR939987. Twelve identical *ITS1* sequences of *Trichinella britovi* were submitted to GenBank under accession number PP153335.

## Supplementary Materials

The following supporting information can be downloaded at: https://www.mdpi.com/article/10.3390/vetsci11020085/s1, Table S1: The primer pairs used for *Trichinella* species identification; Table S2: PCR cycling conditions for *Trichinella* species identification.
