# Supplementary material for: Identification of Sarcocystis and Trichinella Species in Muscles of Gray Wolf (Canis lupus) from Lithuania
Source: Vet Sci. 2024 Feb 10;11(2):85. doi: 10.3390/vetsci11020085 (PMC10892562; doi:10.3390/vetsci11020085)
Supplement: Supplementary file 1 [file vetsci-11-00085-s001.zip › vetsci-2822659-supplementary.pdf]

Table S1. The primer pairs used for *Trichinella* species identification.

ES5 genetic regions	5'-GTTCCATGTGAACAGCAGT-3'
	5'-CGAAAACATACGACAACTGC-3'
ITS1 genetic regions	5'-GCTACATCCTTTTGATCTGTT-3'
	5'-AGACACAATATCAACCACAGTACA-3'

Table S2. PCR cycling conditions for *Trichinella* species identification.

**Temperature, °C**	**Time**	
95	5 min	
94	45 s	5 cycles
58	45 s	
68	1 min	
94	45 s	30 cycles
58	45 s	
72	1 min	
72	5 min	
